# Supplementary material for: Spatial Variation in Stream-Water Bacterial Communities Across Sites Differing in Wild Chinese Giant Salamander Detection Frequency
Source: Biology (Basel). 2026 Sep 18;15(18):1647. doi: 10.3390/biology15181647 (PMC13604818; doi:10.3390/biology15181647)
Supplement: Supplementary file 1 [file biology-15-01647-s001.zip › Data Related to “Spatial Variation in Stream-Water Bacterial Communities across Sites Differing in Wild Chinese Giant Salamander Detection Frequency”/Microbial Data/result/08.FunctionPrediction/picrust2/heatmap/sample/heatmap.sample.level3.cluster.pdf]

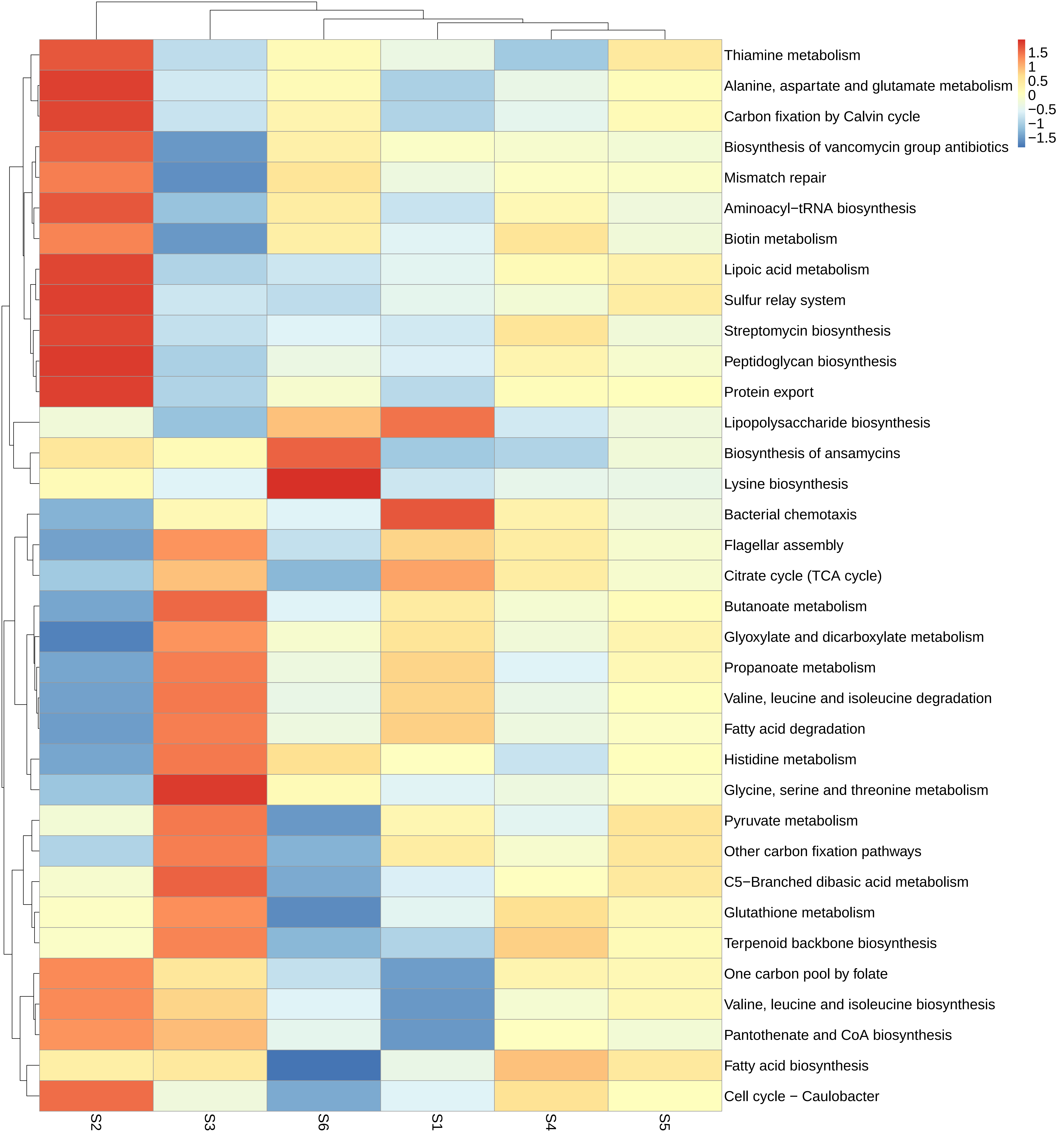
1.5
1
0.5
0
−0.5
−1
−1.5
Thiamine metabolism
Alanine, aspartate and glutamate metabolism
Carbon fixation by Calvin cycle
Biosynthesis of vancomycin group antibiotics
Mismatch repair
Aminoacyl−tRNA biosynthesis
Biotin metabolism
Lipoic acid metabolism
Sulfur relay system
Streptomycin biosynthesis
Peptidoglycan biosynthesis
Protein export
Lipopolysaccharide biosynthesis
Biosynthesis of ansamycins
Lysine biosynthesis
Bacterial chemotaxis
Flagellar assembly
Citrate cycle (TCA cycle)
Butanoate metabolism
Glyoxylate and dicarboxylate metabolism
Propanoate metabolism
Valine, leucine and isoleucine degradation
Fatty acid degradation
Histidine metabolism
Glycine, serine and threonine metabolism
Pyruvate metabolism
Other carbon fixation pathways
C5−Branched dibasic acid metabolism
Glutathione metabolism
Terpenoid backbone biosynthesis
One carbon pool by folate
Valine, leucine and isoleucine biosynthesis
Pantothenate and CoA biosynthesis
Fatty acid biosynthesis
Cell cycle − Caulobacter
S2
S3
S6
S1
S4
S5
